# The Effects of Anti-retroviral Therapy on Epigenetic Age Acceleration Observed in HIV-1-infected Adults

**DOI:** 10.20411/pai.v5i1.376

**Published:** 2020-10-22

**Authors:** Mary E. Sehl, Tammy M. Rickabaugh, Roger Shih, Otoniel Martinez-Maza, Steve Horvath, Christina M. Ramirez, Beth D. Jamieson

**Affiliations:** 1 Department of Medicine, Division of Hematology-Oncology, David Geffen School of Medicine, UCLA; 2 Department of Computational Medicine, David Geffen School of Medicine, UCLA; 3 Department of Pediatrics, Division of Hematology-Oncology, David Geffen School of Medicine, UCLA; 4 Department of Obstetrics and Gynecology, David Geffen School of Medicine, UCLA; 5 Department of Human Genetics, David Geffen School of Medicine, UCLA; 6 Department of Biostatistics, Jonathan and Karin Fielding School of Public Health, UCLA; ŧ These authors contributed equally to this work.

**Keywords:** HIV, aging, methylation, epigenetics, epigenetic clock

## Abstract

**Background:**

HIV-1 infection is associated with acceleration of age-related methylation patterns in peripheral blood and brain of infected individuals although the relative contributions of HIV-1 infection versus its treatment to the observed accelerations in biological aging have not yet been investigated.

**Methods:**

In this longitudinal study of the effects of antiretroviral therapy (ART) on epigenetic aging patterns, we extracted DNA from peripheral blood mononuclear cells from 15 HIV-1-infected individuals infected at three time points: 6 months-1year pre-ART, 6-12 months post-initiation of ART, and 18-24 months after initiating ART. We compared these trajectories with those of 15 age-matched uninfected control participants at three time points with similar intervals. Methylation studies were performed using the Infinium methylation 450 arrays. We examined four epigenetic clock measurements: Age acceleration residual (AAR), Extrinsic (EEAA), Phenotypic (PEAA), and Grim (GEAA) epigenetic age acceleration. Weighted correlation network (WGCNA) analysis was used to identify clusters of highly co-methylated CpGs.

**Results:**

We found that prior to the initiation of ART all four epigenetic measures were significantly higher in HIV-1-infected individuals compared with uninfected individuals (*P<*0.001 for AAR, *P*=0.008 for EEAA, *P*=0.012 for GEAA, *P*<0.001 for PEAA using Wilcoxon rank sum tests between serostatus groups). These effects persisted after the initiation of ART, although the magnitude of these differences diminished. At 18-24 months post-ART initiation (time point 3), PEAA and GEAA were no longer significantly different between HIV-1-infected and uninfected individuals (*P*=0.059 for PEAA, *P*=0.11 for GEAA), while AAR and EEAA remained significantly higher in HIV-1-infected individuals compared with uninfected individuals. We further examined for global patterns of methylation differences between HIV-1-infected and uninfected at each time point, and found 14 groups of co-methylated CpGs that were significantly different between groups at baseline, and remained different after the initiation of ART. Conclusion: We confirm that epigenetic age acceleration associated with HIV-1 infection is most dramatic before ART initiation, and this observation is consistent across four epigenetic clock measurements, as well as in additional groups of co-methylated CpGs identified using WGCNA. Following initiation of ART, there is a partial reduction in age acceleration in all measures, with loss of any significant difference in PEAA and GEAA between serostatus groups. Our findings support the need for future studies examining for a link between epigenetic age acceleration and clinical outcomes in HIV-1-infected individuals.

## INTRODUCTION

Despite dramatic improvements in lifespans for treated virologically suppressed HIV-infected adults, these individuals continue to experience a reduced healthspan compared to their uninfected peers. Treated HIV-infected adults experience earlier decreases in physical functions such as gait-speed [1] and grip strength [2], as well as increased rates of cardiovascular disease, diabetes, osteoporosis, renal failure, liver cancer and neurological dysfunction [3, 4], often a decade earlier than expected.

The mechanisms underlying this shortened healthspan are unclear and no study has yet untangled the effects of HIV infection versus ART [5]. While several studies have confirmed accelerations in age-associated methylation patterns in the peripheral blood of individuals infected with HIV-1 [6–9], previous studies have included only cross-sectional investigations comparing HIV-1-infected and uninfected populations [6], as well as investigations of infected individuals receiving therapy [8].

It is critical to determine whether the epigenetic changes observed in HIV-1-infected individuals are caused by the virus or by antiretroviral therapy, or even both. In the current study, using stored, viably-preserved, peripheral blood samples from the Multicenter AIDS Longitudinal Cohort Study (MACS), we examined longitudinal changes in global methylation patterns of a cohort of men infected with HIV-1, with measurements taken for each participant prior to the initiation of ART by 6 months to 1 year (Visit 1), 6-12 months after initiating ART (Visit 2), and 18-24 months after the initiation of ART (Visit 3). We compared these longitudinal measurements to those of uninfected age-matched control participants at three time points spaced at similar intervals and taken during similar calendar years. By examining multiple epigenetic clocks, as well as groups of co-methylated CpGs associated with both advancing age and HIV-1 infection, we confirm that HIV-1 infection accelerates epigenetic aging, and we observe that ART does not fully restore epigenetic aging to patterns consistent with chronologic age by two years post-ART initiation.

## METHODS

### Ethics Statement

This study was approved by the University of California, Los Angeles Medical Institutional Review Board and each participant was provided written, informed consent per the approved protocol-IRB# 10-001677.

### Participants

Viably cryopreserved peripheral blood mononuclear cell (PBMC) samples were selected from 30 participants enrolled in the Multicenter AIDS Cohort Study (MACS), a study of the natural and treated history of HIV-1 infection in men who have sex with men [10]. There were two groups of participants: 15 HIV-infected individuals (HIV+), 39-48 years of age, who initiated ART while enrolled in the MACS. ART initiation was self-reported, but verified by observed reductions of viral load to less than 50 copies of viral RNA per ml of plasma in all visits selected post ART initiation. Fifteen uninfected individuals (HIV-), age-matched to the HIV+ men, 39-48 years of age, were also matched to the HIV+ men for visit date, history of smoking, and ethnicity. For the HIV+ individuals, we obtained samples 6-12 months pre-ART, 6-12 months post-ART, and 18-24 months post-ART, and corresponding samples from the same visit dates were selected from the HIV-men for a total of 90 samples.

### DNA Isolation

Cryopreserved human peripheral blood mononuclear cells, obtained from the UCLA Multi-Center AIDS Cohort Study (MACS) repository were thawed and assayed for viability using trypan blue. The mean viability of the samples was 88%. A total of 1x106 viable PBMC were used for genomic DNA isolation using Qiagen DNeasy blood and tissue mini spin columns. The quality of DNA samples was assessed using nanodrop measurements and accurate DNA concentrations were measured using a Qubit assay kit (Life Technology).

### Methylation Arrays

Methylation levels at more than 450K CpGs were measured using the Illumina Infinium Human Methylation 450 arrays. These arrays interrogate methylation sites covering 99% of RefSeq genes with an average of 17 CpG sites throughout the promoter 5' and 3' UTRs and coding regions of each gene. In addition, the arrays cover CpG islands, island shores, and other sites distributed throughout the genome. Genomic DNA was prepared as described above. Each DNA sample from an HIV+ individual had a matched sample from an HIV-control individual that was placed on the same chip. Within each chip, the samples were arranged so that HIV+ samples were not placed in adjacent spots with their matched HIV-controls and one of each type of sample on the chip occupied each corner.

Bisulfite conversion was performed by the UCLA Neuroscience Genomics Core facilities. 500 ng of genomic DNA was bisulfate converted using the EZ-methylation kit (Zymo Research, Orange, CA, USA) followed by microarray hybridization of the Human Infinium methylation 450 arrays (Illumina, San Diego, CA), and scanning (iScan, Illumina), according to the manufacturer's protocols by applying standard settings. DNA methylation levels (β values) were determined by calculating the ratio of intensities between methylated (signal A) and unmethylated (signal B) sites. Specifically, the β value was calculated from the intensity of the methylated (M corresponding to signal A) and unmethylated (U corresponding to signal B) sites, as the ratio of fluorescent signals β=Max(M,0)/[Max(M,0) + Max(U,0) + 100]. Thus, β values range from 0 (completely unmethylated) to 1 (completely methylated). To impute missing β values, we used a Euclidean metric to find k-nearest neighbors and impute the missing elements by averaging non-missing elements of its neighbors, using the impute.knn function in R [11]. Quantile normalization was applied to the raw data, in order to detect and remove outliers, and with the goal of making data comparable to the training data of the epigenetic clock.

### Weighted correlation network analysis for finding co-methylated CpG groups

To examine for global methylation changes associated with age, HIV, and ART initiation, we used weighted correlation network analysis (WGCNA) [12]. We used a signed weighted correlation network leading to groups of co-methylated CpGs comprised of positively related CpGs, based on prior work showing that CpGs with a positive age relationship have a different biological interpretation than negatively correlated CpGs [13]. We defined co-methylated CpG groups using average linkage hierarchical clustering [14, 15], and the dynamic hybrid branch cutting approach implemented in the R package dynamicTreeCut [12]. Because each co-methylated CpG group brings together highly correlated methylation profiles, it is useful to summarize the profiles in each GpG group using a single representative profile. We calculated a representative of each co-methylated CpG group (co-methylated CpG group eigenvector), correlated co-methylated CpG group eigenvectors with HIV-1 infection status and ART, and defined a continuous measure of co-methylated CpG group membership (kME, the correlation of the methylation profile with the co-methylated CpG group eigenvector) in the consensus co-methylated GpG group. Defining co-methylated GpG group membership as correlation allows one to easily calculate the statistical significance (P-value) and efficiently annotate all 450,000 CpGs on the Infinium methylation 450 arrays with respect to co-methylated GpG group membership [16].

### Epigenetic Clock Analysis

Here we use the epigenetic clock method (based on the DNAm levels of 353 CpGs) [17]. The method applies to three commercially standardized methylation platforms: the Illumina 850K (EPIC), 450K and 27K arrays. The epigenetic clock method is an attractive biomarker of aging because (1) it applies to most human tissues; and (2) its accurate measurement of chronological age is unprecedented [17]. Furthermore, it is accelerated in disease states and is predictive of all-cause mortality after adjusting for known risk factors [18]. Predicted age, referred to as DNAm age, correlates with chronological age in sorted cell types (CD4 T cells, monocytes, B cells, glial cells, neurons) and tissues and organs including whole blood, brain, breast, kidney, liver, lung, and saliva [17]. An online age calculator can be found at our webpage (https://dnamage.genetics.ucla.edu/new).

We examined four measures of epigenetic age acceleration: Age acceleration residual (AAR) based on 353 CpGs of Horvath [17], Extrinsic epigenetic age acceleration (EEAA) based on 71 CpGs of Hannum [19], Phenotypic epigenetic age acceleration (PEAA) based on 513 CpGs of Levine [20], and Grim epigenetic age acceleration (GEAA) based on 1030 CpGs of Lu [21]. Briefly, AAR is a multi-tissue clock that captures epigenetic age acceleration and is valid for a wide range of tissue types. It is highly correlated with chronological age and accelerated in disease states. While both PEAA and GEAA predict lifespan (time-to-death and all-cause mortality), PEAA was developed by regressing a phenotypic measure of mortality risk on CpGs, whereas GEAA is formed by regressing time-to-death on DNAm-based surrogate biomarkers of smoking pack-years and a selection of plasma proteins previously associated with mortality or morbidity [20, 21]. Note that here we do not examine intrinsic epigenetic age acceleration, which is based on the Horvath clock with adjustment for cell composition, because HIV is known to affect cell composition. By construction, EEAA is positively correlated with senescent T lymphocytes and negatively correlated with naïve T lymphocytes.

### Statistical Analysis of Epigenetic Clocks

DNA methylation (DNAm) age was estimated using the online epigenetic clock software (http://dnamage.genetics.ucla.edu) which calculates Horvath's pan tissue clock, Grim Age, Phenotypic Age, blood cell counts and many additional DNAm based biomarkers [17]. The epigenetic “age acceleration residual” (AAR, defined as the residual regressing DNAm age on chronologic age) was compared between HIV-infected and uninfected individuals, using the Wilcoxon rank sum test, a non-parametric group comparison test, at each of three visits (one prior to, and two following the initiation of antiretroviral therapy). By definition, chronological age is not correlated (r=0) with our measures of epigenetic age acceleration (AAR, EEAA, PEAA, GEAA).

We examined between group differences in means for AAR, EEAA, PEAA, and GEAA in HIV-infected compared with HIV-uninfected individuals using the non-parametric Wilcoxon rank-sum test at all three time points studied. In order to test for within-group differences from Visit 1 to Visit 2, from Visit 1 to Visit 3, and Visit 2 to Visit 3, we examined the Paired Samples Wilcoxon signed-rank test. All tests were two-sided.

### Weighted Gene Co-methylation Network Analysis:

While the epigenetic clock is a robust and highly revealing measurement of DNAm age, it is restricted to the 353 CpG sites previously shown to be tightly correlated with chronologic age across multiple cell and tissue types [17]. Similarly, the EEAA, PEAA, and GEAA measures are restricted to the 71, 513, 1,030 CpGs, respectively, from which they are constructed. Variables such as age and HIV-1 infection will also influence thousands of other CpG sites throughout the genome [6]. The wider influence of these variables on the epigenome can be explored through the application of weighted gene co-expression network analysis (WGCNA) [12].

A signed weighted correlation network analysis was used to identify co-methylated CpG groups comprised of positively related CpGs. After identifying these co-methylated CpG groups, we calculated a representative of each co-methylated CpG group (co-methylated CpG group eigenvector), and examined whether each co-methylated CpG group eigenvector was associated with HIV infection or antiretroviral therapy use, using the Kruskal Wallis non-parametric multi group comparison test.

For co-methylated CpG groups identified as significantly associated with either HIV infection or antiretroviral therapy use, we examined between group differences in mean co-methylated CpG group eigenvector methylation in HIV-infected compared with HIV-uninfected individuals using the non-parametric Wilcoxon rank-sum test at all three study Visits. In order to test for within group differences from Visit 1 to Visit 2, from Visit 1 to Visit 3, and Visit 2 to Visit 3, we examined the Paired Samples Wilcoxon signed-rank test.

## RESULTS

### AAR and EEAA, but not GEAA and PEAA, remain significantly higher in HIV-1-infected compared with uninfected individuals after the initiation of ART

Table 1 describes the clinical characteristics of subjects at study visits. ART initiation occurred in HIV-1-infected subjects between Visit 1 and Visit 2. The average age±SD at ART initiation was 43.7±3.3 years. In order to examine whether epigenetic age is accelerated in the setting of HIV-1 infection in the absence of therapy, and whether this acceleration persists after the introduction of therapy, we examined four measures of DNA methylation (AAR, EEAA, PEAA, and GEAA) at three time points: 6 months to 1 year pre-ART (Visit 1), 6-12 months after the initiation of ART (Visit 2), and 18-24 months after the initiation of ART (Visit 3). When we compare HIV-1-infected seropositive men at their pre-ART visit with seronegative men, we find that untreated HIV-1-infected men had a DNAm age that was approximately 8.4 years “older” than their chronologic age and significantly more advanced than their uninfected peers (*P*=<0.001) (see Figure 1A). Despite ART initiation and successful suppression of HIV-1-RNA to less than 50 copies per ml of plasma, at approximately 2 years post-ART initiation, the HIV+ men demonstrated a DNAm age that was approximately 6.8 years “older” than their chronologic age and these men remained significantly “older” than their uninfected peers *(P*=0.017), documenting that by 2 years post-ART initiation ART was unable to fully restore their DNAm age to patterns consistent with their chronologic age. Also of interest, is our finding that the HIV-men also appear to be slightly older by DNAm age than their chronologic age by an average of 2 years at baseline (range: −4.3, 9.5, *P*=0.06 by the signed rank test), although this is within the range of normal variation of this measure [17].

**Table 1.** Characteristics of subjects at study visits.

	HIV-1-infected (N=15)			Uninfected (N=15)		
Characteristics at Visit	Visit 1	Visit 2	Visit 3	Visit 1	Visit 2	Visit 3
Age (years), Mean ± SD	42.9 ± 3.3	44.7 ± 3.4	45.7 ± 3.3	42.6 ± 3.5	44.9 ± 3.4	45.8 ± 3.3
CD4 T cells (cells/mL), Mean ± SD	353 ± 190	480 ± 221	535 ± 245	970 ± 331	960 ± 338	966 ± 395
CD8 T cells (cells/mL), Mean ± SD	1,124 ± 598	1,069 ± 457	995 ± 556	492 ± 131	498 ± 162	435 ± 157
HIV viral load (copies/mL)^a^, Mean ± SD	41,456 ± 67,317	< 50 ± 0	< 50 ± 0			

a ART initiation in HIV-1-infected subjects occurred between Visit 1 and Visit 2

**Figure 1A.**
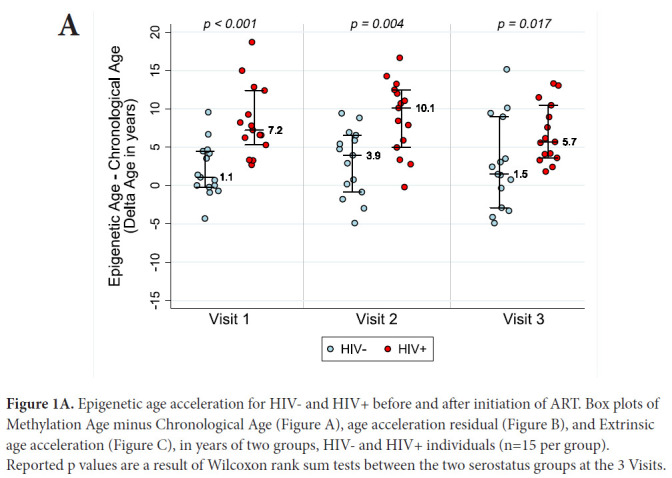
Epigenetic age acceleration for HIV- and HIV+ before and after initiation of ART. Box plots of Methylation Age minus Chronological Age (Figure A), age acceleration residual (Figure B), and Extrinsic age acceleration (Figure C), in years of two groups, HIV- and HIV+ individuals (n=15 per group). Reported p values are a result of Wilcoxon rank sum tests between the two serostatus groups at the 3 Visits.

AAR, calculated using the residuals obtained when regressing DNAm age on chronologic age, was significantly higher in HIV-1-infected individuals compared with matched HIV-controls (*P*<0.001 using a nonparametric Wilcoxon rank sum test comparing median values). AAR was significantly higher in HIV-1-infected HIV+ compared with controls at both 6-12 months (*P*=0.003) and 18 months to 2 years (*P*=0.019) post-ART initiation (see Figure 1B) despite successful viral suppression.

**Figure 1B & C.**
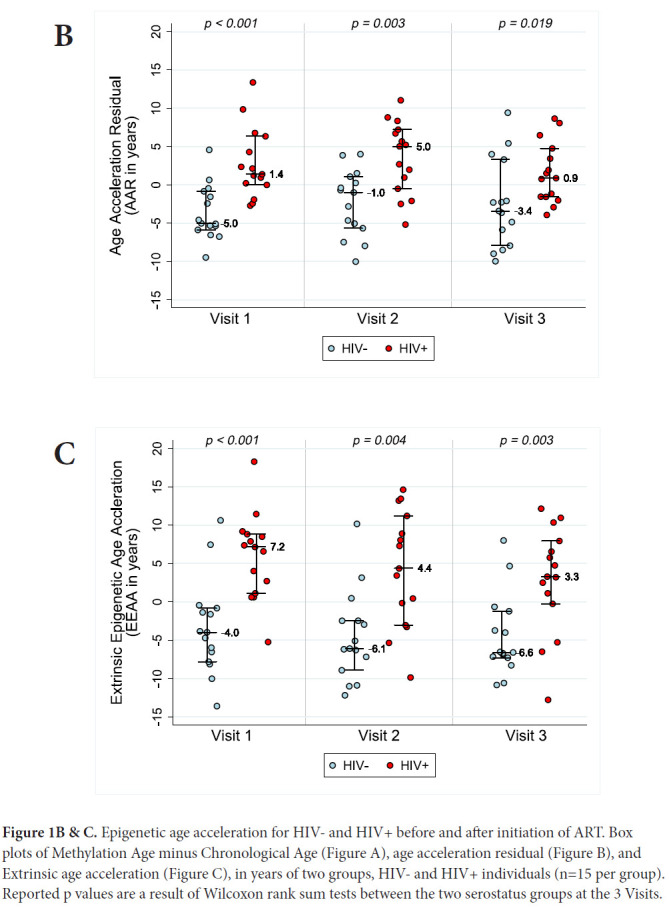
Epigenetic age acceleration for HIV- and HIV+ before and after initiation of ART. Box plots of Methylation Age minus Chronological Age (Figure A), age acceleration residual (Figure B), and Extrinsic age acceleration (Figure C), in years of two groups, HIV- and HIV+ individuals (n=15 per group). Reported p values are a result of Wilcoxon rank sum tests between the two serostatus groups at the 3 Visits.

Extrinsic age is based on 71 CpGs [19] and is positively, and negatively, correlated with senescent and naïve cytotoxic T cells, respectively. Figure 1C reveals EEAA for HIV- and HIV+ individuals at each visit. This measure is significantly higher in HIV+ individuals compared with HIV-individuals (*P*<0.001 using the Wilcoxon rank sum test) at Visit 1. The mean difference in EEAA between HIV- and HIV+ groups decreases after initiation of ART but the HIV+ group exhibits significantly higher degrees of acceleration at both 6-12 months post-ART and 18-24 months post-ART *(P=*0.003).

**Figure 2A & B.**
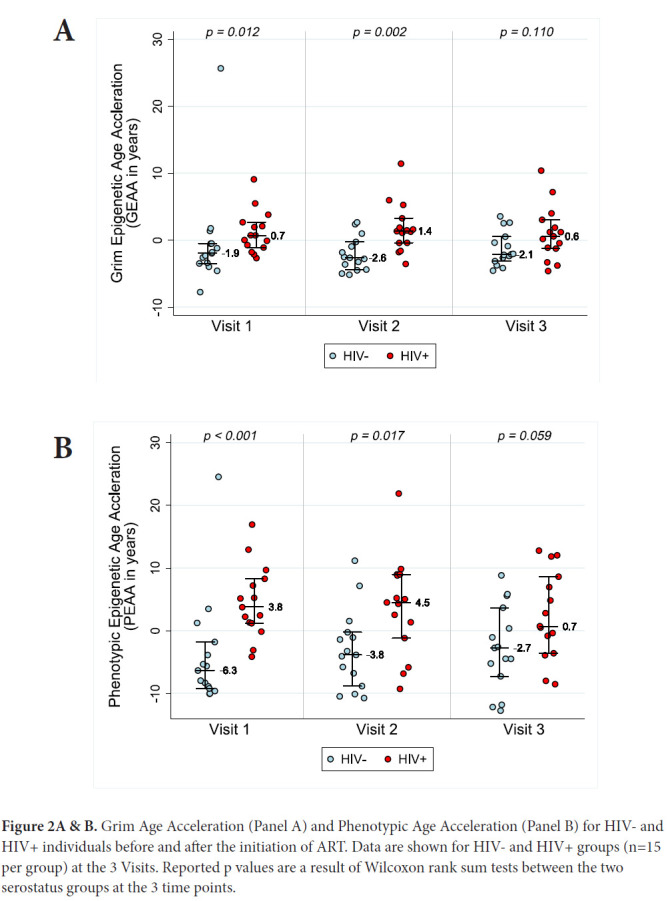
Grim Age Acceleration (Panel A) and Phenotypic Age Acceleration (Panel B) for HIV- and HIV+ individuals before and after the initiation of ART. Data are shown for HIV- and HIV+ groups (n=15 per group) at the 3 Visits. Reported p values are a result of Wilcoxon rank sum tests between the two serostatus groups at the 3 time points.

In order to assess acceleration in epigenetic measures of healthspan, morbidity, and mortality risk, we examined both PEAA and GEAA. Both Phenotypic age, based on 513 CpGs [20], and Grim age [21], based on 1030 CpGs, are highly predictive of lifespan and healthspan. In addition, Grim age is associated with clinical biomarkers and age-related medical conditions [21]. We set out to examine whether these measures were also accelerated in HIV-1 infection. Figure 2 reveals GEAA (Panel 2A) and PEAA (Panel 2B) for HIV- and HIV+ individuals before and after the initiation of ART therapy. With these two clocks, we detect the largest mean difference between HIV- and HIV+ groups prior to the initiation of ART *(P=*0.012 for GEAA, *P*<0.001 for PEAA). At 18-24 months after the initiation of ART, there is no significant difference between HIV- and HIV+ in both GEAA *(P=*0.11) and PEAA *(P=*0.059).

### Global methylation patterns associated with HIV-1 infection are largely unaffected by ART

We applied WGCNA to explore the effects of HIV-1 infection and ART on changes in the degree of methylation at CpG sites throughout the more than 450,000 CpG sites assessed in the methylation array. We identified 83 clusters of CpGs whose methylation levels were highly correlated in our analysis compared with a single reference co-methylated CpG group. Of these,14 co-methylated CpG group eigenvectors were significantly associated with HIV-1 infection status, using a Bonferroni correction of 0.05/83=0.0006.

**Figure 3.**
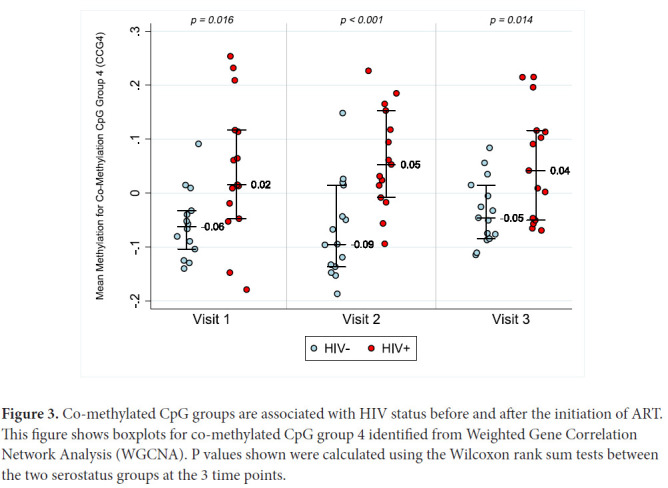
Co-methylated CpG groups are associated with HIV status before and after the initiation of ART. This figure shows boxplots for co-methylated CpG group 4 identified from Weighted Gene Correlation Network Analysis (WGCNA). P values shown were calculated using the Wilcoxon rank sum tests between the two serostatus groups at the 3 time points.

In order to examine whether global methylation differences between HIV- and HIV+ individuals change with ART, we compared mean methylation for each co-methylated CpG group eigenvector (CCG) at each time point. CCG4, our largest co-methylated CpG group with 41,384 CpG sites, is a representative example of a co-methylated CpG group associated with HIV-infection, and this association did not appear to be influenced by the initiation of ART (Figure 3). Other co-methylated CpG groups showed similar patterns, consistent with a lack of global ART-induced changes in HIV-1-induced methylation patterns.

### Polycomb Group Target genes (PcGT) are highly represented in Co-methylated CpG group 4

Co-methylated CpG group 4 (CCG4) was the largest of the 14 co-methylated CpG groups. Using the online functional annotation tool David (https://david-d.ncifcrf.gov/), we analyzed the CpGs contained within CCG4 for their gene ontology revealing that within CCG4 41,384 CgG sites were associated with 30,682 genes. To better analyze the data, we pared the CpG sites down to those 2,581 CpG that had a kME of >0.85. Those sites were then grouped by DAVID into 997 gene clusters, only 273 of which met the Benjamini cutoff of *P*>0.05. Supplementary Table 1 lists the Top 49 gene pathways that these 273 genes fall into.

The co-methylation CpG group identified in our global analysis (CCG4) is enriched for CpGs that fall within genes associated with the polycomb group target (PCGT) pathway, which is involved in stem cell self-renewal, and has shown to be altered in aging, dysplasia, and carcinogen-esis. This analysis was performed to search for pathways, in addition to those within the Horvath, Hannum, Grim, and Phenotypic epigenetic aging clocks, that could be associated with aging, HIV, and ART initiation.

Polycomb Group Target Genes (PcGT) are involved in the regulation of development, maintenance of progenitor cell identity, and cancer [22]. We previously reported that the co-methylated CpG group most highly correlated with both untreated HIV-1 status and aging, co-methylation CpG group 3 (CCG3, previously published under the term “module eigenvector 3”), had 14 PcGT that fell among the 990 genes with a strong co-methylated CpG group membership as defined by a kME of >0.85 [6]. In the study reported herein, all but one of those PcGT genes (FBX039) were also found in CCG4, demonstrating overlap of gene clusters between our current CCG4 and our previously reported CCG3 [6]. As in our previous study [6], these genes had multiple CpGs that fell within CCG4, suggesting strong suppression of those genes in response to HIV-1 infection and minimal to no change with ART initiation. The presence of these genes is associated with development, stemness, and cancer, and the presence of multiple CpG sites for each gene within CCG4 suggests this co-methylated CpG group captures genes with a functional relationship with aging. The overlap of these genes between two separate studies demonstrates the reproducibility of our results across disparate samples and datasets.

## DISCUSSION

We have confirmed that age-associated methylation patterns are accelerated in HIV-1-infected men, and this accleration is present before the start of antirretroviral therapy. We find that four measures of biological age-acceleration: AAR, EEAA, PEAA, and GEAA are elevated in HIV-1 infection prior to the initiation of ART, clearly demonstrating the role of HIV infection in biologic age acceleration. We further show that ART partially reduces the degree of acceleration of all of these measures of biological aging, but not to the baseline uninfected levels for AAR and EEAA. Even in the setting of adequate therapy there is still an ongoing acceleration of epigenetic aging according to all four epigenetic clocks.

While the magnitude of the mean difference in epigenetic age acceleration decreases for each of the four clocks from pre- to post-treatment, we find that for two measures based on Horvath's pan tissue clock (AAR and intrinsic epigenetic age acceleration) there is a persistently significant difference between HIV- and HIV+ individuals at all visits. By contrast, for GEAA and PEAA, the difference between HIV- and HIV+ individuals is not significantly different at Visit 3 (18-24 months after the initiation of ART), which probably reflects the normalization of blood-cell counts following successful treatment. Unlike the Horvath pan tissue clock, Grim age, Phenotypic age, and the Hannum age estimators exhibit substantially stronger correlations with blood-cell counts. This finding suggests that epigenetic measures that are highly predictive of lifespan and healthspan (Grim age and Phenotypic age) improve with ART earlier than epigenetic clocks that are more reflective of properties of hematopietic stem cells (such as the Horvath pan tissue clock). These observations are consistent with the observation that even several years after the initiation of ART, HIV-1-infected adults experience increased morbidity from age-related illnesses compared with HIV-1-uninfected individuals, as well as increased frailty, geriatric syndromes, and non-AIDS related cancers including lung cancer [4, 23–25].

Our findings raise the question of whether epigenetic aging mediates age-related diseases such as those found in treated HIV-infected individuals. They further raise the question of whether accelerated biologic aging can be reversed and how fast we might expect possible reversal of epigenetic aging to affect the progression of these processes.

Also of interest is the relationship between immune system composition, well documented to change during aging and HIV infection, and epigenetics. We hypothesize that immunosenescence represents at least one mechanism through which accelerated epigenetic aging may contribute to mortality in HIV infection. Immunosenescence has been shown to be associated with both faster HIV-1 disease progression, [26] dysregulated immunity, and chronic diseases in HIV-1-infected adults [27], leading others to conclude that immunosenescence is a contributor to accelerated aging in HIV-infected adults [28]. We previously documented a strong association between accelerated epigenetic age, as measured by the epigenetic clock, and activated and senescent CD8+ T-cells [6]. Those results were again found in this study (data not shown). Future studies could be directed towards identifying individuals at risk for early onset of age-related illnesses, and consideration of senolytic therapies as potential prevention for age-related illnesses in older individuals living with HIV. Focused interventions and preventive strategies, such as exercise interventions, could be directed towards those individuals identified, using the epigenetic clock, to exhibit a high degree of accelerated biological aging and at higher risk of earlier onset of age-related illnesses.

The CpG groups identified in the global analysis are enriched for CpGs that fall within genes associated with the polycomb group target (PCGT) pathway, which is involved in stem cell self-renewal, and has shown to be altered in aging, dysplasia, and carcinogenesis. This analysis was performed to search for pathways, in addition to those within the Horvath, Hannum, Grim, and Phenotypic epigenetic aging clocks, that could be associated with aging, HIV, and ART initiation. While the pan-tissue Horvath clock, Phenotypic, and Grim aging clocks are associated with PCGT protein target sites, the Grim and Phenotypic aging clocks are further enriched for markers of inflammation. These include pathways involved in pro-inflammatory signaling, multiple toll-like receptor signaling, the JAK-STAT cascade, TNF-mediated signaling, and NFκB transcription factor activity. In addition, acceleration of the Phenotypic age clock is inversely associated with expression of genes associated with DNA damage recognition and repair.

There are several limitations with this study, the first being the small sample size. Larger samples sizes will be needed to confirm that the GEAA and PEAA are restored faster than the AAR and EEAA. While our HIV-1-infected and uninfected participants were matched on age, we were unable to adjust our analyses for additional covariates because of our limited sample size. Future larger studies are needed to examine the effects of ART on epigenetic aging, with adjustment for key demographic and clinical covariates including ethnicity, body mass index, and coexisting illnesses. Another limitation is that we did not rigorously investigate the introduction of new epi-genetic changes by ART, which could affect the “restoration” of the different clocks. This will also be important to assess for future studies that may wish to explore associations between post-ART epigenetic patterns and future clinical outcomes.

We find that, at 18-24 months after ART initiation, there is no significant difference between epigenetic age acceleration, as measured by PhenoAge and GrimAge, between HIV-1-infected and uninfected individuals. Because HIV-1-infected adults experience increased earlier morbidity from age-related illnesses compared with HIV-1 uninfected adults after initiation of ART, we would expect epigenetic age acceleration to persist in all measures, particularly those most closely related to morbidity and mortality. However, we do note that AAR and EEAA remain persistently different long after ART initiation, suggesting that the epigenetic mechanisms captured by these clocks may drive HIV-associated age-related morbidity that continues after treatment. Larger studies are needed to assess whether acceleration in epigenetic aging is associated with outcomes such as age-related illnesses and frailty.

## Figures and Tables

**Table:** Supplementary Table 1.

Term	Count	%	Benjamini
Developmental protein	116	14.8	1.0E-40
Homeobox, conserved site	70	6.2	2.1E-34
Homeobox	77	6.9	4.4E-33
Homeodomain	76	6.8	1.2E-30
DNA-binding region:Homeobox	65	5.8	3.8E-30
HOX	76	6.8	2.8E-25
Homeodomain-like	79	7.0	1.2E-24
Homeodomain, metazoa	42	3.7	2.2E-24
Sequence-specific DNA Binding	99	8.8	3.8E-23
Cell adhesion	87	7.8	1.5E-21
DNA-binding	215	19.2	6.2E-21
Glycoprotein	370	33.0	8.2E-18
Glycosylation site:N-linked (GlcNAc…)	357	31.8	5.4E-17
Positive regulation of transcription from RNA polymerase II promoter	135	12.0	1.0E-16
Domain:Cadherin 5	36	3.2	6.5E-16
Cadherin	38	3.4	8.3E-16
Transcription regulation	219	19.5	9.2E-16
Cadherin conserved site	37	3.3	1.1E-15
Cadherin-like	38	3.4	1.1E-15
Domain:Cadherin 1	37	3.3	1.6E-15
Domain:Cadherin 2	37	3.3	1.6E-15
Transcription	222	19.8	2.2E-15
Homophilic cell adhesion via plasma membrane adhesion molecules	45	4.0	3.6E-15
Domain:Cadherin 3	36	3.2	4.0E-15
Domain:Cadherin 4	36	3.2	4.0E-15
Topological domain:Cytoplasmic	293	26.1	2.1E-13
Calcium	106	9.4	2.3E-13
Negative regulation of transcription from RNA polymerase II promoter	103	9.2	2.8E-13
Cell adhesion	77	6.9	3.1E-12
Transcriptional activator activity, RNA polymerase II core promoter proximal region sequence-specific binding	51	4.5	3.2E-13
transcription factor activity, sequence-specific DNA binding	120	10.7	4.3E-13
CA	38	3.4	5.9E-13
Domain:Cadherin 6	29	2.6	7.1E-13
Cadherin, N-terminal	26	2.3	7.6E-13
Alternative splicing	686	61.1	3.1E-12
Signal peptide	279	24.9	1.0E-11
Topological domain:Extracellular	241	21.5	2.1E-11
RNA polymerase II core promoter proximal region sequence-specific DNA binding	60	5.3	4.6E-11
Neurogenesis	45	4.0	9.9E-11
Cell junction	82	7.3	2.4E-10
Transcription from RNA polymerase II promoter	76	6.8	3.2E-10
Synapse	54	4.8	4.8E-10
Compositionally biased region:Poly-Ala	60	5.3	1.2E-09
Calcium ion binding	90	8.0	1.2E-09
Anterior/posterior pattern specification	26	2.3	1.9E-09
Signal	308	27.5	6.2E-09
Nervous system development	50	4.5	1.2E-08
Cell membrane	245	21.8	2.2E-08
Synapse	37	3.3	2.5E-08

**Table:** Supplementary Table 2.

CpG	kME42	GeneName
cg13122498	0.774737673	BTBD9;BTBD9;BTBD9
cg08418670	0.760519061	SH3GL1
cg07141605	0.732497633	
cg08782022	0.72872463	MGRN1;MGRN1;MGRN1;MGRN1
cg15401418	0.72387172	SEPT9;SEPT9;SEPT9;SEPT9
cg07804973	0.692108311	TFDP1;TFDP1
cg01785719	0.67539606	TSNARE1
cg02097604	0.666707573	TOM1L2;TOM1L2
cg19628469	0.666440823	ELANE
cg10953317	0.662354955	CD300A
cg07159958	0.660151102	ATL1;ATL1;ATL1
cg24138325	0.643920669	ARHGEF10
cg03071500	0.643656651	
cg14191616	0.635103246	SH3RF3
cg10979567	0.632899349	COL6A3;COL6A3;COL6A3
cg01528913	0.624226268	
cg13492600	0.622277247	RFX2;RFX2
cg18769421	0.613724466	COBRA1
cg20102280	0.61211928	HTR2A;HTR2A
cg03773198	0.611724352	FHAD1
cg03916490	0.609177299	C7orf50;C7orf50;C7orf50
cg24079038	0.608562626	KCNQ1
cg10317778	0.601817618	
cg00499781	0.601026822	A2BP1;A2BP1;A2BP1;A2BP1;A2BP1;A2BP1
cg02893779	0.595505185	SETD3;SETD3
cg24591969	0.595489187	KCNAB2;KCNAB2
cg23476885	0.591684881	MCCC1
cg27554875	0.591113665	ZCCHC5
cg23096377	0.587538299	C7orf50;C7orf50;C7orf50
cg26058778	0.585351093	
cg12059309	0.58505386	CNOT2
cg10258241	0.575100154	
cg15464485	0.574893617	C7orf50;C7orf50;C7orf50
cg11282828	0.574560752	
cg08702344	0.568745676	CLDN12
cg23238058	0.561738011	
cg18709570	0.556136406	
cg03978067	0.550097998	EEF1D;EEF1D;EEF1D;EEF1D;EEF1D;EEF1D;EEF1D
cg05315334	0.549314772	MCF2L;MCF2L
cg07083806	0.547327562	MYCN;MYCNOS
cg00124375	0.543421721	DDAH2
cg03848483	0.541210415	
cg09329826	0.538461625	PDZD4
cg24536250	0.534971915	
cg01620164	0.534936861	FIGN
cg17360854	0.534425664	RBMS1;RBMS1
cg14249520	0.532872377	CPT1A;CPT1A
cg09422696	0.532780782	
cg20409752	0.531258247	CAMTA1
cg24697097	0.530702267	
cg22708738	0.529329286	FUZ
cg05648303	0.518800369	SCOC;SCOC;SCOC;SCOC;SCOC
cg03075605	0.514322979	JRKL;JRKL;CCDC82
cg00236832	0.49687569	RARA;RARA
cg14181940	0.487635848	CD300A
cg26988138	0.485510319	GNG7
cg03130891	0.483769664	
cg13989519	0.483267333	TSPAN18
cg04910877	0.477383373	CBFA2T3;CBFA2T3
cg23052855	0.475060162	ZDHHC19
cg04705659	0.469605512	PLEKHA6
cg26802063	0.463863482	ARSB;ARSB;ARSB
cg23889772	0.463786601	COL5A3
cg25875683	0.452337816	PLXNB3;PLXNB3;PLXNB3;PLXNB3
cg05513908	0.438472341	C22orf36;GGT1
cg06873401	0.432684492	LHFPL2
cg05656566	0.432667807	ADAMTS2;ADAMTS2
cg19544767	0.43188012	
cg22504140	0.424587486	
cg21786191	0.423815267	ZADH2
cg06654691	0.423231877	PLEKHH3
cg05085169	0.41836259	OGFR
cg03079733	0.415564378	RIPK4
cg00186529	0.415220033	
cg19092368	0.383563039	CTPS
cg05105330	0.363560798	KIAA0513
cg25013753	0.360060327	ARHGAP22
cg14781919	0.355498045	ITGAE;GSG2
cg23664926	0.354744948	
cg03589751	0.327483304	KIAA1199;MIR549
cg10772532	0.319667132	C14orf145;C14orf145
cg18553732	0.305635755	CHKA;CHKA
